# Expanding the phenotypic spectrum of *NOTCH1* variants: clinical manifestations in families with congenital heart disease

**DOI:** 10.1038/s41431-024-01629-4

**Published:** 2024-05-22

**Authors:** Kaitlin J. Stanley, Kelsey J. Kalbfleisch, Olivia M. Moran, Rajiv R. Chaturvedi, Maian Roifman, Xin Chen, Roozbeh Manshaei, Nicole Martin, Simina McDermott, Vanda McNiven, Diane Myles-Reid, Lynne E. Nield, Miriam S. Reuter, Marci L. B. Schwartz, Patrick Shannon, Rachel Silver, Cherith Somerville, Ronni Teitelbaum, Laura Zahavich, Anne S. Bassett, Raymond H. Kim, Seema Mital, David Chitayat, Rebekah K. Jobling

**Affiliations:** 1 Ted Rogers Centre for Heart Research Cardiac Genome Clinic, The Hospital for Sick Children, Toronto, ON Canada; 2 Division of Clinical and Metabolic Genetics, Department of Pediatrics, The Hospital for Sick Children, Toronto, ON Canada; 3 Division of Cardiology, Department of Pediatrics, The Hospital for Sick Children, Toronto, ON Canada; 4 The Prenatal Diagnosis and Medical Genetics Program, Department of Obstetrics and Gynecology, Mount Sinai Hospital, Toronto, ON Canada; 5 Division of Genetics, Department of Pediatrics, McMaster Children’s Hospital, Hamilton, ON Canada; 6 The Centre for Applied Genomics, The Hospital for Sick Children, Toronto, ON Canada; 7 Genetics and Genome Biology Program, The Hospital for Sick Children, Toronto, ON Canada; 8 Department of Pathology and Laboratory Medicine, Mount Sinai Hospital, Toronto, ON Canada; 9 The Dalglish Family 22q Clinic, University Health Network, Toronto, ON Canada; 10 Department of Psychiatry, University of Toronto, Toronto, ON Canada; 11 Clinical Genetics Research Program and Campbell Family Mental Health Research Institute, Centre for Addiction and Mental Health, Toronto, ON Canada; 12 Fred A. Litwin Family Centre in Genetic Medicine, Department of Medicine, University Health Network, Toronto, ON Canada; 13 Ted Rogers Centre for Heart Research, Toronto, ON Canada; 14 Genome Diagnostics, Department of Pediatric Laboratory Medicine, The Hospital for Sick Children, Toronto, ON Canada

**Keywords:** Health care, Diseases

## Abstract

Pathogenic variants in *NOTCH1* are associated with non-syndromic congenital heart disease (CHD) and Adams–Oliver syndrome (AOS). The clinical presentation of individuals with damaging *NOTCH1* variants is characterized by variable expressivity and incomplete penetrance; however, data on systematic phenotypic characterization are limited. We report the genotype and phenotype of a cohort of 33 individuals (20 females, 13 males; median age 23.4 years, range 2.5–68.3 years) from 11 families with causative *NOTCH1* variants (9 inherited, 2 de novo; 9 novel), ascertained from a proband with CHD. We describe the cardiac and extracardiac anomalies identified in these 33 individuals, only four of whom met criteria for AOS. The most common CHD identified was tetralogy of Fallot, though various left- and right-sided lesions and septal defects were also present. Extracardiac anomalies identified include cutis aplasia (5/33), cutaneous vascular anomalies (7/33), vascular anomalies of the central nervous system (2/10), Poland anomaly (1/33), pulmonary hypertension (2/33), and structural brain anomalies (3/14). Identification of these findings in a cardiac proband cohort supports *NOTCH1*-associated CHD and *NOTCH1*-associated AOS lying on a phenotypic continuum. Our findings also support (1) Broad indications for *NOTCH1* molecular testing (any familial CHD, simplex tetralogy of Fallot or hypoplastic left heart); (2) Cascade testing in all at-risk relatives; and (3) A thorough physical exam, in addition to cardiac, brain (structural and vascular), abdominal, and ophthalmologic imaging, in all gene-positive individuals. This information is important for guiding the medical management of these individuals, particularly given the high prevalence of *NOTCH1* variants in the CHD population.

## Introduction

The human *NOTCH1* gene encodes a 300 kDa transmembrane receptor protein, Notch1, that activates the Notch signaling pathway [1, 2]. The Notch signaling pathway is highly conserved and plays an essential role in developmental processes such as vasculogenesis, cardiac embryogenesis, and primordial valve formation [1–6]. Deleterious variants in *NOTCH1* are known to influence these processes and are involved with several congenital disorders.

It is well-established that haploinsufficiency of *NOTCH1* causes aortic valve disease (MIM # 109730). There is considerable interfamilial and intrafamilial variability in the *NOTCH1* cardiac phenotype described in the literature, including left-sided lesions (e.g., bicuspid aortic valve and hypoplastic left heart (HLH)), right-sided lesions, conotruncal defects, and septal defects (Supplementary Table 1) [2, 4, 6–19]. Recently, the increased use of exome sequencing and genome sequencing (GS) has demonstrated that *NOTCH1* variants are a more frequent cause of congenital heart disease (CHD) than was previously recognized, particularly in the context of tetralogy of Fallot (TOF), where deleterious *NOTCH1* variants have been deemed responsible for 4–5% of cases [15, 20, 21].

Heterozygous pathogenic variants in *NOTCH1* can also cause autosomal dominant Adams–Oliver syndrome (AOS; MIM # 616028) [5, 6, 17, 22, 23]. Current evidence supports the idea of AOS being a primary defect of vasculogenesis [6, 24, 25]. AOS is characterized by cutis aplasia involving the scalp, terminal transverse limb defects, and other variable vascular abnormalities, including pulmonary and portal hypertension (Table 1) [17, 22, 23]. A clinical diagnosis of AOS is given when criteria involving major clinical features, outlined by Lehman et al. (2016) [26], are met.

**Table 1:** Summary of the features described in cohorts of *NOTCH1*-related Adams–Oliver syndrome and in our cohort.

AOS feature	Previous citing literature		Total in literature	Total in cohort
	Stittrich et al. [23]	Southgate et al. [17]		
TTLD	6	13	19	0
Cutis aplasia	5	13	18	5
Cardiac malformation	3	8	11	27
Bony skull defect		7	7	1
Cutis marmorata	4	2	6	3
Hypoplastic/aplastic nails	5	1	6	1
Brachydactyly	3	1	4	3
Toe hypoplasia	2	1	3	0
Syndactyly	2	1	3	0
Intracranial vascular lesions	3		3	4
Portal hypertension		3	3	0
Long palpebral fissures		2	2	0
Down-slanting palpebral fissures		1	1	1
Hypertelorism		1	1	1
White vesicles at fingertips	1		1	0
Portal vein hypoplasia	1		1	1
Tortuous scalp vessels	1		1	0
Hemangioma	1		1	0
Pulmonary hypertension	1		1	2
Hernia		1	1	1
Cryptorchidism		1	1	0
Lymphopenia		1	1	0
Myopathy		1	1	0
Epilepsy		1	1	3
Intellectual disability	1		1	0
Learning disability		1	1	3
Autism		1	1	0
Spastic diplegia	1		1	0

*AOS* Adams–Oliver syndrome, *TTLD* terminal transverse limb defect.

There is no clear genotype-phenotype correlation that dictates whether an individual with a deleterious *NOTCH1* variant will develop isolated CHD or AOS [6, 11, 15]. To assess the proposal that *NOTCH1*-associated CHD and *NOTCH1*-associated AOS represent a continuous spectrum of clinical findings [6, 15], and to examine the interfamilial and intrafamilial variability, we present a cohort of individuals and their families who were ascertained through a proband with CHD and found to have a causative *NOTCH1* variant. The clinical relevance of the extracardiac phenotypes observed highlight the importance of *NOTCH1* genetic testing for ongoing patient care. Using these results and the existing literature, we propose recommendations for cascade testing and surveillance of individuals with *NOTCH1* variants. This is vital as an increasing number of gene-positive individuals are ascertained through their CHD phenotypes.

## Subjects & methods

### Ethical considerations

This study was approved by the Research Ethics Board (REB) at The Hospital for Sick Children (SickKids), Toronto, Ontario, Canada (REB# 1000053844) and the University Health Network, Toronto, Ontario Canada (REB# 16-6282) All participants, or their substitute decision-makers, provided written informed consent to participate in the Cardiac Genome Clinic (CGC) study protocol [27, 28].

### Participants & identification of *NOTCH1* variants

This was a cohort study of individuals with *NOTCH1* variants and their families. All probands were initially ascertained because of a CHD diagnosis. Probands (*N* = 11) and their relatives (*N* = 22) with *NOTCH1* variants were identified as candidates for this case series either by their clinical geneticist, or by screening of the CGC research database (*N* = 512 families with GS data) of patients recruited from cardiology clinics at SickKids, University Health Network, or Mount Sinai Hospital. *NOTCH1* variants were identified in one of three ways: (1) Through research GS involving the proband conducted by the CGC (methods referenced in [27, 28]); (2) Through clinically indicated genetic testing (e.g., a gene panel or single gene test) for the proband; or (3) Through cascade testing in at-risk relatives, following identification of a *NOTCH1* variant in a proband by the aforementioned methods. Research GS results were clinically validated in an approved clinical laboratory. Table 2 outlines the testing method used for each proband.

**Table 2:** NOTCH1 variants identified in the 11 families studied.

Family	Ethnicity	Testing method for proband	*NOTCH1* variant	Interpretation	Rationale documenting clinical relevance using ACMG criteria
**A**	Native Canadian	Research GS: proband + affected sibling.	127 kbp deletion 9q34.3-9q34.3 (encompassing entire *NOTCH1* gene and no other OMIM morbid map genes)	Pathogenic	**Variant previously reported:** Similar deletion reported in Kerstjens-Frederikse et al. [13]. ACMG criteria (copy number LOSS): **2A** - Complete overlap of haploinsufficient gene NOTCH1 (+1.00).
**B**	European	Research GS: trio	c.13_14dupCT p.Ala6Trpfs*28	Likely pathogenic	**Variant previously reported:** No ACMG criteria **PVS1 –** Frameshift, where LOF is a known mechanism of disease. **PM2 –** Absent from gnomAD.
**C**	European	Clinical *NOTCH1* sequencing: singleton + research GS: quad (in parallel)	c.2995G>A p.Val999Met	VUS	**Variant previously reported:** ClinVar ID: 1036675. Family previously reported in Gordon et al. [31]. ACMG criteria: **PM2 –** Present at low frequency in controls (total AF = 0.00001474; maximal AF = 0.00008253 in South Asian subpopulation). **PP1** – Segregates with CHD in 3 family members. **PP2 –** *NOTCH1* is intolerant to missense variation.
**D**	African/European	Research GS: proband + affected sibling.	c.141-1G>C p.?	Likely pathogenic	**Variant previously reported:** No ACMG criteria: **PVS1** – Variant is in the canonical splice site, where LOF is a known mechanism of disease. **PM2 –** Absent from gnomAD
**E**	European/Native Canadian	Research GS: proband + 3 affected relatives	c.568C>T p.Arg190Cys	VUS	**Variant previously reported:** No ACMG criteria: **PM2 –** Present at low frequency in controls (total AF = 0.000001429; maximal AF = 0.000001852 in Non-Finnish European subpopulation) **PP1** – Segregates with CHD in 4 family members. **PP2 –** *NOTCH1* is intolerant to missense variation.
**F**	European	Clinical *NOTCH1* sequencing: singleton + research GS: trio (in parallel)	c.5814C>G p.Tyr1938*	Likely pathogenic	**Variant previously reported:** No ACMG criteria: **PVS1** – Nonsense variant, where LOF is a known mechanism of disease. **PM2 –** Absent from gnomAD.
**G**	African/European/Middle Eastern	Clinical ES: trio	c.3654T>A p.Cys1218*	Pathogenic	**Variant previously reported:** No ACMG criteria: **PVS1** – Nonsense variant, where LOF is a known mechanism of disease. **PS2 –** De novo variant where parentage is confirmed. **PM2 –** Absent from gnomAD.
**H**	Eastern European/Ashkenazi Jewish	Clinical AOS gene panel: singleton	c.4415G>A p.Cys1472Tyr	Likely pathogenic	**Variant previously reported:** No. ACMG criteria: **PM2 –** Absent from gnomAD. **PM5 –** Another variant at the same amino acid (p.Cys1472Trp) is reported as likely pathogenic in Alankarage et al. [32]. **PP1 (Moderate)** – Segregates with CHD/cutis aplasia in 4 family members. **PP2 –** *NOTCH1* is intolerant to missense variation. **PP3** – Variant is predicted damaging by all in silico tools (CADD = 26.7; REVEL = 0.836)
**I**	European	Clinical AOS gene panel: singleton	c.4579C>T p.Gln1527*	Pathogenic	**Variant previously reported:** No ACMG criteria: **PVS1** – Nonsense variant, where LOF is a known mechanism of disease. **PM2 –** Absent from gnomAD. **PP1_Strong:** Previously reported to segregate with disease.
**J**	European	Research GS: trio	c.866-2A>G p.?	Likely pathogenic	**Variant previously reported:** No ACMG criteria: **PVS1** – Variant is in the canonical splice site, where LOF is a known mechanism of disease. **PM2 –** Absent from gnomAD
**K**	European	Research GS: trio	c.5349del p.Arg1784Glyfs*14	Likely pathogenic	**Variant previously reported:** No ACMG criteria: **PVS1** – Nonsense variant, where LOF is a known mechanism of disease. **PM2 –** Absent from gnomAD.

Transcript referenced is NM_017617. Variants were interpreted using guidelines outlined by the American College of Medical Genetics (ACMG) [29, 30]. Allele frequencies referenced are from gnomAD v4.1.0. The level of evidence supporting segregation data (PP1) was adjusted based on guidelines from Jarvik and Browning (2016) [43].

*AF* allele frequency, *AOS* Adams–Oliver syndrome, *CHD* congenital heart disease, *ES* exome sequencing, *gnomAD* genome aggregation database, *GS* genome sequencing, *LOF* loss of function, *VUS* variant of uncertain significance.

### Variant assessment

*NOTCH1* variants were classified according to the variant interpretation guidelines outlined by the American College of Medical Genetics [29, 30].

### Clinical investigations

After identification of the *NOTCH1* variant, a retrospective chart review was conducted for each participant. Gene-positive individuals had a targeted work-up directed by their clinical geneticist. This work-up included, where possible and if not previously done/available, a physical exam, echocardiogram, brain magnetic resonance imaging (MRI) and angiography (MRA), ophthalmologic assessment, and abdominal ultrasound with Doppler imaging of the liver and kidneys. Clinical information about extended family members who were not genotyped was included by report only.

## Results

### *NOTCH1* variants

Thirty-three individuals (11 probands, 8 parents, and 14 other relatives, including one obligate carrier) were found to have a clinically relevant *NOTCH1* variant. Six families (A, C, D, E, F, H, and I) had more than one affected relative at the time of ascertainment; Proband F-III:1 had a brother with a right aortic arch, who later tested negative for the familial *NOTCH1* variant. Two families (B and G) had negative family histories and de novo *NOTCH1* variants were identified. Two families (J and K) had no known family history at ascertainment, but cascade testing and subsequent clinical assessment revealed other affected relative(s).

Table 2 summarizes the testing methodology used to identify the 11 rare *NOTCH1* variants included, as well as their classification. Nine of these variants were classified as pathogenic or likely pathogenic (Families A, B, D, F, G, H, I, J, and K), and two were classified as variants of uncertain significance (VUS) but are clinically suspicious and believed to be excellent candidates given the family history and their segregation with disease (Families C and E). Two variants were confirmed de novo and inheritance was unknown for one. Nine of the 11 variants were absent from gnomAD v4.1.0. A similar deletion to the one in Family A was previously published in Kerstjens-Frederikse et al. [13], Family C was previously published in the Gordon et al. [31] cohort, and a similar variant to the one in Family H was previously reported by Alankarage et al. [32].

In those individuals who had GS, additional rare damaging variants were identified but none were diagnostic (Supplementary Table 2).

Eighteen additional rare *NOTCH1* VUS (including one variant found in two reportedly unrelated families) were identified by screening of the CGC database but excluded from this analysis due to insufficient evidence of pathogenicity (e.g., relatively high allele counts in gnomAD, low in silico pathogenicity predictions, lack of segregation, poor phenotypic fit, and/or alternative molecular diagnoses to explain the proband’s CHD) (Supplementary Table 3).

### Demographics

Twenty females and 13 males are described. The median age of the 29 living individuals was 23.4 years (range 2.5–68.3 years) at the time of chart review. The remaining four participants include two fetuses (post-termination of pregnancy), one female who died at 6 months of age due to post-operative pulmonary hypertensive arteriopathy, and one male who died at age 36 following a cardiac procedure.

### Clinical features

**Fig. 1:**
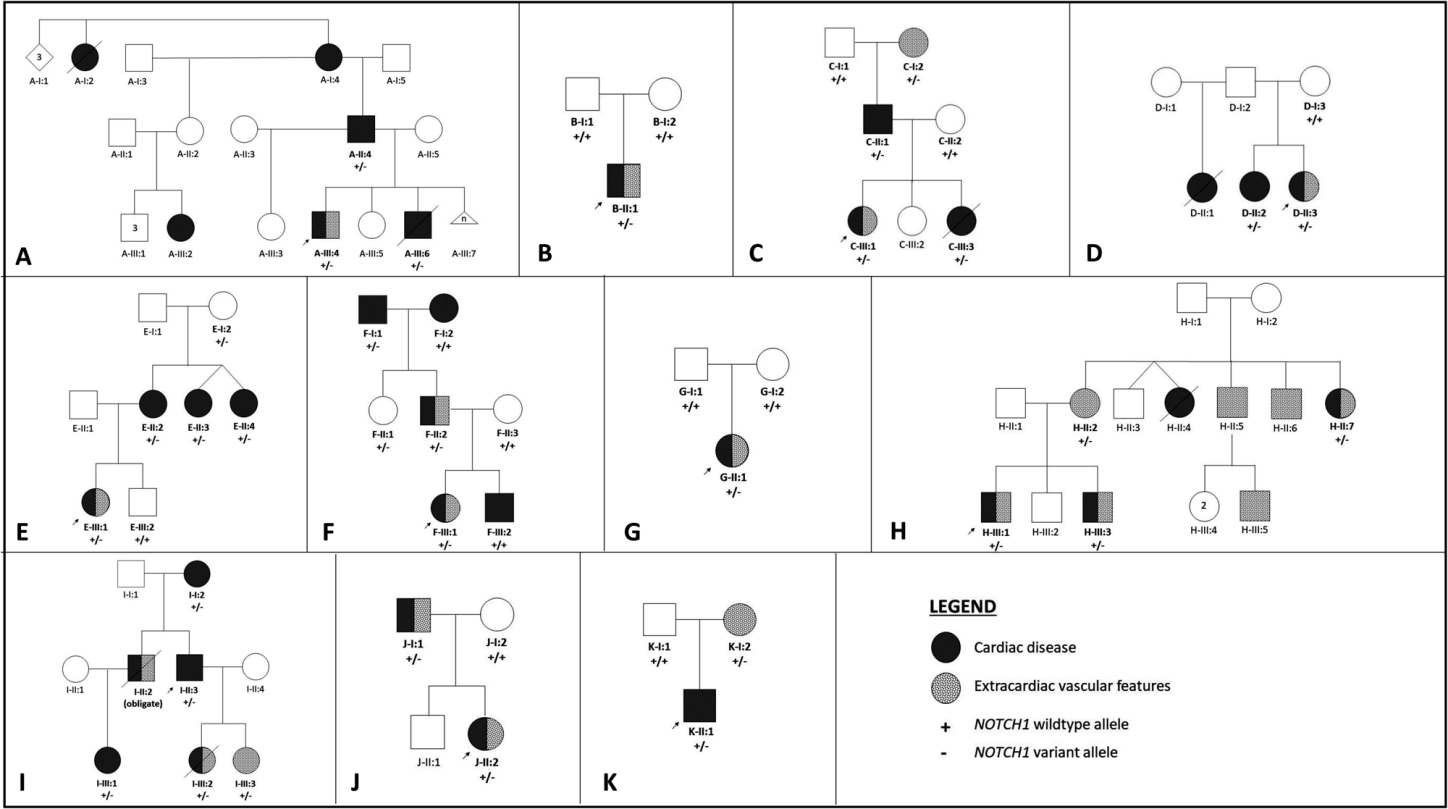
Pedigree structures of families with identified *NOTCH1* variants, ascertained through a proband with congenital heart disease. **A**–**K** are labeled for the family name referenced in the text (e.g., Family A is depicted in (**A**)). Probands are marked by a black arrow. The genetic status of all 47 individuals who had genetic testing is included, with “+” denoting the *NOTCH1* wildtype allele and “−” denoting the *NOTCH1* variant allele. Individuals shaded in solid black had clinically known, structural cardiac disease (confirmed by review of echocardiogram where possible). Individuals shaded in hatched grey had extracardiac vascular features.

All clinical details are in Supplementary Table 4, and pedigrees are in Fig. 1. Two individuals (E-I:2 and F-II:1) had no documented cardiac or extracardiac features. As shown in Fig. 1, there were additional relatives who had genetic testing and were negative for the familial *NOTCH1* variant; two of whom had CHD that are relatively common in the general population (F-I:2 had a bicuspid aortic valve and F-III:2 had a right aortic arch).

#### Cardiac features

Of the 33 individuals described, 27 (all 11 probands and 16 relatives) had a documented structural cardiac anomaly. The most common CHD in our cohort was TOF (present in nine individuals from six families). Of the six individuals with no documented cardiac disease, four had a normal echocardiogram and two are pending echocardiogram.

Discovery of the familial *NOTCH1* variant changed cardiac care for five individuals. Two individuals (I-I:2, I-II:3) had previously known cardiac pathology but only started receiving regular cardiac care after their genetic diagnosis, with one individual (I-I:2) subsequently requiring an aortic valve replacement. Another three individuals (F-I:1, F-II:2, J-I:1) received cardiac diagnoses and began anticipatory care after their *NOTCH1* diagnosis prompted an echocardiogram.

#### Extracardiac features

**Fig. 2:**
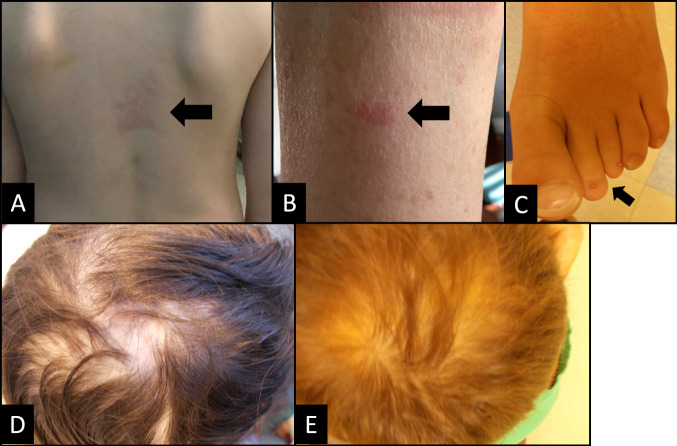
Images depicting the extracardiac features of select individuals with *NOTCH1* variants. **A** Cutaneous vascular malformation on the lower back of individual B-II:1. **B** Cutaneous vascular malformation on the forearm of individual C-I:2. **C** Toe brachydactyly and hypoplastic nails on individual H-III:1. **D** Cutis aplasia on the scalp of individual H-II:2. **E** Cutis aplasia on the scalp of individual H-III:3.

**Fig. 3:**
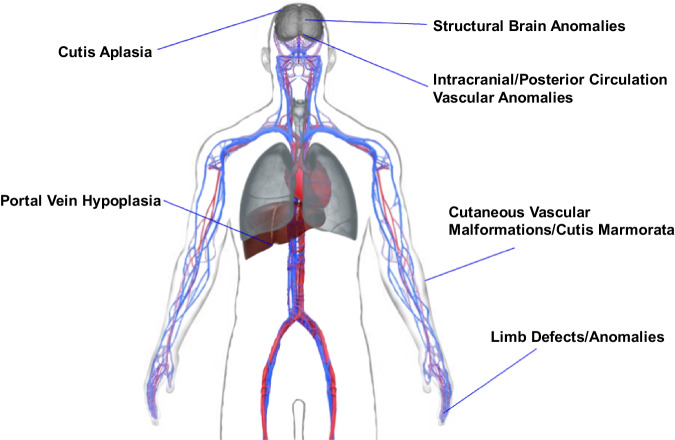
The extracardiac phenotype of individuals with a clinically relevant *NOTCH1* variant, based on results from the cohort studied. Features include cutis aplasia involving the scalp, structural brain anomalies, intracranial/posterior circulation vascular anomalies, portal vein hypoplasia, cutaneous vascular malformations (including cutis marmorata), and limb anomalies.

Figures 2 and 3 depict some of the extracardiac features identified in our cohort. Only four individuals in the cohort (H-III:1, H-III:3, H-II:2, and I-III:3) met clinical diagnostic criteria for AOS upon identification of their *NOTCH1* variant.

##### Vascular anomalies

Of the 12 individuals who underwent brain MRA, posterior circulation abnormalities were identified in two individuals. One had severe bilateral hypoplasia of the vertebrobasilar artery system (D-II:2), requiring ASA prophylaxis and ongoing neurology follow-up for transient visual changes and headaches related to vertebrobasilar insufficiency. The other, who has passed away, was found to have a hypoplastic right vertebral artery (I-III:2).

Of the nine individuals who had ophthalmologic assessments, one (I-III:3) was found to have tortuous retinal blood vessels at 2.6 years of age, requiring lifelong monitoring for retinal hemorrhage.

On Doppler examination of the portal veins, G-II:1 had a hypoplastic portal venous system with nonspecific slow flow in the left portal vein. Of the eight other individuals who had Doppler examination of the liver, no anomalies were identified other than fatty infiltration or fibrosis. These can occur secondary to congestive hepatopathy caused by elevation of systemic venous pressures in patients after certain types of CHD surgery.

Cutis marmorata was observed in three individuals; an additional two individuals reported mottling. Two individuals had cutaneous vascular malformations (Fig. 2A, B).

Other clinically significant abnormalities included bilateral pulmonary arteriovenous malformations that may have been secondary to the underlying cardiac disease (B-II:1), congenital absence of the right pectoralis muscle, consistent with Poland anomaly (F-II:2), a large left middle cerebral artery ischemic infarct and, on autopsy, widespread pulmonary hypertensive arteriopathy, beyond what was expected by the extent and nature of the pulmonary thrombo-emboli present (I-III:2), and pulmonary hypertension (J-I:1).

##### Central nervous system anomalies

Of the 17 individuals that had a brain MRI, three had structural anomalies. F-III:1 exhibited a diffusely small corpus callosum and prominent lateral ventricles. This individual also had slightly reduced cerebral white matter volume consistent with prior insult. F-II:2 had a focus of subependymal grey matter heterotropia in the left frontal region. G-II:1 had microcephaly (head circumference 32 cm, corresponding to the second percentile for age and sex-matched controls), enlarged peri-cerebral extra-axial spaces and lateral ventricles, a thin corpus callosum, delayed myelin maturation, and other findings in keeping with prior infarcts.

Three individuals had a history of seizures, one with a diagnosis of treatment-responsive epilepsy (A-III:4), one with a single seizure in the context of an abnormal brain MRI (G-II:1), and one with subclinical seizures based on abnormal electroencephalogram (I-III:2). Additionally, there were five individuals with reported history of developmental delay or learning disability.

##### Limb anomalies

None of the 33 individuals described had terminal transverse limb defects; however, several did have digit differences, including brachydactyly of the fingers or toes, or clinodactyly. H-III:3 exhibited toe brachydactyly with hypoplastic nails (Fig. 2C).

##### Cutis aplasia

Cutis aplasia was confirmed in four individuals from two families (Fig. 2D, E) and was suspected in one individual with scalp bald spots.

##### Other

H-III:1 had a solitary right kidney, and H-III:3 had a bony skull defect (parietal bone abnormality). F-III:1 had hypertension of unknown etiology. Many individuals had other congenital and/or pediatric-onset conditions requiring care, such as scoliosis, hernias, and cysts.

## Discussion

Our results highlight the broad indications for testing *NOTCH1* in individuals with CHD. The described individuals presented with structural cardiac anomalies, with both interfamilial and intrafamilial variability. A recent study by Debiec et al. [33] suggests analysis of *NOTCH1* in all sporadic and familial cases of both TOF and HLH and consideration in cases of familial bicuspid aortic valve and associated simple CHD (aortic coarctation and ventricular septal defect). We would agree that an expanded consideration of the indications for *NOTCH1* testing is warranted given the variability associated with this genotype and its frequency in the CHD population. It is notable that one of the families reported here (Family A) had a history of only septal defects prior to ascertainment through an individual with TOF. Our findings support consideration of *NOTCH1* analysis in any case of familial CHD, in addition to simplex cases of TOF and HLH. Furthermore, our identification of a *NOTCH1* variant in several seemingly unaffected family members highlights the value of cascade testing in all at-risk relatives.

Several individuals described also have a wide range of extracardiac features that have not been previously characterized in a CHD cohort. Transverse terminal limb defects, a characteristic feature of AOS, were not observed in our cohort, which is unsurprising since its presence would have prompted an assessment for AOS in infancy. Cutis aplasia congenita, cutis marmorata, and the digit anomalies observed in our cohort are known features of AOS and have been observed specifically in the context of *NOTCH1* variants [17, 23, 26]. The bony skull defect (parietal bone abnormality in H-III:3), intracranial vascular lesions (vertebrobasilar artery stenosis in D-II:3, and hypoplastic right vertebral artery in I-III:2), portal vein hypoplasia (G-II:1), abnormal neurodevelopmental trajectories, and seizures described in our cohort have also been previously associated with *NOTCH1*-associated AOS [17, 23, 26]. There are also features we observed that were previously reported in AOS families without genotype data in the older literature, such as liver fibrosis and brain malformations, including, ventriculomegaly, corpus callosum dysgenesis, and delayed myelin maturation [26].

Considering the essential role of *NOTCH1* in vasculogenesis, which is a crucial step in the embryogenesis of all body organs, the posterior circulation anomalies, brain anomalies, and portal vein hypoplasia observed in our cohort may be related to the *NOTCH1* variants. Variable arterial anomalies are a well-described feature of AOS, so the vertebrobasilar artery anomalies found in two of our patients are likely attributable to *NOTCH1* haploinsufficiency [26]. Some brain abnormalities seen in our cohort, such as ventriculomegaly and delayed myelination, are observed more frequently in individuals with severe CHD and may be a consequence of the CHD itself [34–37]. Other anomalies we observed, including the small corpus callosum and heterotopia, have not been clearly associated with CHD. It is also worth noting that small/hypoplastic corpus callosum and ventriculomegaly have been observed in individuals with AOS in the absence of severe CHD [26, 38]. Similarly, while portal hypertension and liver fibrosis can be seen in individuals with severe CHD [39], hypoplasia of the portal venous system appears to be uncommon. Portal hypertension, hepatoportal sclerosis, and small/absent portal venous system have all been previously reported in AOS [17, 25]. Future case-control studies will be important to assess the frequency of structural brain and portal venous abnormalities features in CHD cohorts of individuals with and without *NOTCH1* variants.

This is the first description of an absent right pectoralis muscle (Poland anomaly) in an individual with a *NOTCH1* variant (F-II:2). The etiology of the Poland anomaly is not well understood, but it is thought to arise from abnormalities during vasculogenesis, resulting in an interruption of the early embryonic blood supply to the affected area [40]. Poland anomaly has been previously reported in two families with AOS, though no genotypes were available [41]. The authors proposed that Poland anomaly and AOS may be variable manifestations of a single dominant gene variant that causes developmental vascular accidents [41, 42], which could be consistent with *NOTCH1* defects. Notably, none of the individuals with *NOTCH1* variants in Family F have a clinical diagnosis of AOS.

The presence of multiple extracardiac findings in a cohort of families ascertained through CHD underscores the need for additional clinical screening when a *NOTCH1* variant is identified. In this cohort, only four individuals (H-III:1, H-III:3, H-II:2, and I-III:3) fulfill diagnostic criteria for AOS [26]. The remaining 29 individuals do not. Those without a diagnosis of AOS do not have defined guidelines for screening and management. Given the established role of *NOTCH1* in vasculogenesis and the high prevalence of extracardiac clinical findings in the current study, we propose that additional screening, as described by Lehman et al. (2016) [26], should be performed in all gene-positive individuals. This includes brain MRI for structural anomalies, MRA of head and neck vessels, abdominal ultrasound with examination of the kidneys and liver, and portal vein assessment. Careful examination of the scalp for mild cutis aplasia, skin examination, and eye exam should also be performed, though at this point vascular anomalies in the eye appear to be rare in individuals with *NOTCH1* variants (present in only one individual in our cohort). Given the challenge of obtaining this level of screening in all individuals, we recommend that a reasonable effort be made based on resources available.

The combination of both cardiac and extracardiac phenotypes associated with *NOTCH1* variants, along with the intrafamilial variability observed, finally brings forth some counselling challenges. For example, Family I has one individual with AOS and several others with non-syndromic CHD. Cases like this make it difficult to accurately discuss phenotypic recurrence and prepare families for the presentation of more, or less, severe phenotypes in future pregnancies. However, this challenge is not unique to *NOTCH1*-related disorder, as genotype-first approaches continue to redefine the phenotypic spectrum of many genetic disorders.

### Limitations

Limitations of the present study include that complete clinical data were not available for all individuals described. Second, given the broad inclusion criteria for the CGC study from which many families were ascertained (which includes CHD, in addition to aortopathy, cardiomyopathy, and arrhythmias), commenting on the prevalence of *NOTCH1* variants in CHD is beyond the scope of this paper. Third, functional studies were not available but may have aided in the classification of the VUS described. Finally, data was not available to compare if the burden of extracardiac findings was higher among individuals with a *NOTCH1* variant to those without.

## Conclusions

We report a cohort of families with a clinically relevant *NOTCH1* variant, initially ascertained for seemingly isolated CHD in a proband. Subsequent cascade testing and clinical evaluation revealed variable cardiac and extracardiac vascular anomalies in the probands and their family members. Our findings support broad indications for *NOTCH1* molecular testing and highlight the importance of cascade testing in all at-risk relatives. Although these probands were ascertained in the context of their CHD, our identification of *NOTCH1* variants in family members without cardiac disease (e.g., in individuals with only cutis aplasia) indicates that the phenotypic spectrum of *NOTCH1* variants extends beyond clinical CHD. Upon identification of a pathogenic *NOTCH1* variant, we propose the implementation of multi-system screening previously recommended for individuals with AOS [26]. This screening is warranted for all gene-positive individuals, even in the absence of a clinical diagnosis of AOS, given the presence of extracardiac anomalies in our CHD cohort. However, further phenotyping of larger cohorts is needed to establish the frequency of cardiac and extracardiac findings in individuals with *NOTCH1* variants compared to those without *NOTCH1* variants and to further refine screening recommendations. Finally, given the presence of these vascular findings in cardiac patients, and the presence of both “conditions” (isolated CHD and AOS) in the same family with the same variant, our observations provide further support that these two diagnoses are within the same phenotypic spectrum of *NOTCH1*-related disorder.

### Supplementary information

Supplementary Table 1

Supplementary Table 2

Supplementary Table 3

Supplementary Table 4

## Data Availability

The data that support the findings of this study are available from the corresponding author upon reasonable request.
