# Supplementary material for: Expanding the phenotypic spectrum of NOTCH1 variants: clinical manifestations in families with congenital heart disease
Source: Eur J Hum Genet. 2024 May 22;32(7):795–803. doi: 10.1038/s41431-024-01629-4 (PMC11219983; doi:10.1038/s41431-024-01629-4)
Supplement: Supplementary file 1 — Supplementary Table 1 [file 41431_2024_1629_MOESM1_ESM.docx]

**Supplementary Table 1:** Summary of the cardiac lesions associated with deleterious *NOTCH1* variants, as described in the literature and in the present study cohort. Abbreviations include: TOF = tetralogy of Fallot; MAPCAs = major aortopulmonary collateral arteries; BAV = bicuspid aortic valve; HLH = hypoplastic left heart; VSD = ventricular septal defect; DORV = double outlet right ventricle; ASD = atrial septal defect; PDA = patent ductus arteriosus; HRH = hypoplastic right heart; AVSD = atrioventricular septal defect; TAPVC = total anomalous pulmonary venous connection.

| Cardiac Lesion | Previous Citing Literature | | | | | | | | | | | | | | **Total in Literature** | **Total in Cohort** |
| --- | --- | --- | --- | --- | --- | --- | --- | --- | --- | --- | --- | --- | --- | --- | --- | --- |
|  | Garg et al. (2005) | McKellar et al. (2007) | McBride et al. (2008) | Wang et al. (2011) | Freylikhman et al. (2014) | Southgate et al. (2015) | Kerstjens-Frederikse et al. (2016) | Durbin et al. (2017) | Zahavich et al. (2017) | Helle et al. (2019) | Page et al. (2019) | Debiec et al. (2020) | Kalayinia et al. (2020) | Roifman et al. (2021) |  |  |
| TOF (+/- MAPCAs) |  |  |  | 1 |  |  | 1 |  |  |  | 37 |  |  | 1 | 40 | 9 |
| BAV | 7 | 5 | 4 |  | 5 | 1 | 12 |  |  | 2 |  | 1 |  | 3 | 40 | 9 |
| Aortic stenosis | 5 |  | 6 |  |  | 3 | 14 |  | 1 |  |  | 2 |  |  | 31 | 6 |
| HLH | 1 |  | 3 |  |  |  |  | 1 | 1 | 10 |  |  |  | 1 | 17 | 4 |
| Aortic calcification | 7 | 2 |  |  |  |  | 1 |  |  | 2 |  | 1 |  | 1 | 14 | 1 |
| Aortic dilatation |  | 5 |  |  |  |  | 4 |  |  |  |  | 2 |  |  | 11 | 2 |
| VSD | 1 |  |  | 1 |  | 2 | 1 |  | 1 | 1 |  | 1 | 1 | 1 | 10 | 5 |
| Hypoplastic aortic arch |  |  |  |  | 7 |  |  |  |  | 1 |  |  |  | 1 | 9 | 1 |
| Aortic coarctation |  |  | 2 |  |  | 2 | 2 |  |  |  |  |  |  | 1 | 7 | 2 |
| Mitral stenosis | 1 |  |  |  |  |  | 3 |  |  |  |  |  |  |  | 4 | 3 |
| DORV | 1 |  |  |  |  |  |  |  | 1 |  |  |  |  | 1 | 3 | 5 |
| Pulmonary atresia |  |  |  |  |  | 1 | 2 |  |  |  |  |  |  |  | 3 | 3 |
| Pulmonary stenosis |  |  |  |  |  |  | 2 |  | 1 |  |  |  |  |  | 3 | 3 |
| Aortic valve regurgitation |  |  |  |  |  | 3 |  |  |  |  |  |  |  |  | 3 | 4 |
| ASD |  |  |  |  |  | 1 | 1 |  |  | 1 |  |  |  |  | 3 | 1 |
| PDA |  |  |  |  |  |  | 2 |  |  |  |  |  | 1 |  | 3 | 1 |
| Truncus arteriosus |  |  |  |  |  | 1 | 2 |  |  |  |  |  |  |  | 3 | 1 |
| Mitral atresia | 1 |  |  |  |  |  |  |  | 1 |  |  |  |  |  | 2 | 4 |
| Asymmetric aortic valve |  |  |  |  |  |  | 2 |  |  |  |  |  |  |  | 2 | 0 |
| Parachute mitral valve |  |  |  |  |  | 2 |  |  |  |  |  |  |  |  | 2 | 0 |
| Dysplastic mitral valve |  |  |  |  | 1 |  |  |  |  |  |  |  |  | 1 | 2 | 0 |
| Coronary artery disease |  |  |  |  |  |  |  |  |  |  |  | 1 |  |  | 1 | 1 |
| HRH |  |  |  |  |  |  | 1 |  |  |  |  |  |  |  | 1 | 0 |
| AVSD |  |  |  |  |  |  |  |  |  |  |  |  | 1 |  | 1 | 0 |
| Bicuspid pulmonary valve |  |  |  |  |  |  |  |  |  |  |  |  |  | 1 | 1 | 0 |
| Tricuspid valve dysplasia |  |  |  |  |  |  |  |  |  |  |  |  |  | 1 | 1 | 0 |
| Pulmonary artery abnormalities |  |  |  |  |  |  |  |  |  |  |  |  |  |  | 0 | 5 |
| Right aortic arch |  |  |  |  |  |  |  |  |  |  |  |  |  |  | 0 | 3 |
| Pulmonary valve regurgitation |  |  |  |  |  |  |  |  |  |  |  |  |  |  | 0 | 2 |
| Aortic atresia |  |  |  |  |  |  |  |  |  |  |  |  |  |  | 0 | 1 |
| Pulmonary valve dysplasia |  |  |  |  |  |  |  |  |  |  |  |  |  |  | 0 | 1 |
| Tricuspid atresia |  |  |  |  |  |  |  |  |  |  |  |  |  |  | 0 | 1 |
| TAPVC |  |  |  |  |  |  |  |  |  |  |  |  |  |  | 0 | 1 |
| Other |  |  |  |  | 2* |  |  |  |  |  |  |  |  |  | 2 | 0 |
| Normal echo |  |  |  |  |  | 5 | 6 | 1 |  |  |  |  |  |  | 12 | 4 |

* Lesions not specified in literature
