# Supplementary material for: Expanding the phenotypic spectrum of NOTCH1 variants: clinical manifestations in families with congenital heart disease
Source: Eur J Hum Genet. 2024 May 22;32(7):795–803. doi: 10.1038/s41431-024-01629-4 (PMC11219983; doi:10.1038/s41431-024-01629-4)
Supplement: Supplementary file 3 — Supplementary Table 3 [file 41431_2024_1629_MOESM3_ESM.docx]

**Supplementary Table 3:** Additional 18 rare *NOTCH1* variants identified in a proband with cardiac disease by screening of the Cardiac Genome Clinic (CGC) research database. One variant was identified in two unrelated families. Rare is defined as a maximal allele frequency (AF) of that variant in any subpopulation in gnomAD v4.1.0 below 0.0005. Transcript referenced is ENST00000277541. These variants were excluded from the present analysis due to insufficient evidence of pathogenicity. In all cases, there were relatively high allele counts in gnomAD, low in silico pathogenicity predictions, lack of segregation within the family, poor phenotypic fit, and/or another molecular diagnosis that explained the proband’s CHD. Abbreviations include: AS = aortic stenosis; BAV = bicuspid aortic valve; CoA = coarctation of the aorta; HCM = hypertrophic cardiomyopathy; HLH = hypoplastic left heart; HRH = hypoplastic right heart; TGA = transposition of the great arteries; TOF = tetralogy of Fallot; VSD = ventricular septal defect.

|  | |  | | |  |  |  |  |  |  |  |  |  |  |
| --- | --- | --- | --- | --- | --- | --- | --- | --- | --- | --- | --- | --- | --- | --- |
| ID | Primary heart lesion | | c. | p. | | Inherited? | SIFT | PolyPhen | BayesDel | ClinPred | CADD | REVEL | PhyloP | Allele frequency in gnomAD v4.1.0 |
| 1 | AS, PS | | c.1934G>A | p.Cys645Tyr | | Yes | deleterious | probably damaging | D | D | 27.4 | 0.93 | 7.74 | 0.000003423 |
| 2 | TGA/VSD, CoA | | c.649C>T | p.Arg217Trp | | Yes | tolerated | possibly damaging | T | T | 24.9 | 0.378 | 1.958 | 0.00001415 |
| 3 | Heterotaxy | | c.2908A>G | p.Thr970Ala | | Yes | tolerated | possibly damaging | T | T | 23.4 | 0.449 | 6.019 | 0.00005766 |
| 4 | CoA | | c.4027G>A | p.Ala1343Thr | | Yes | tolerated | possibly damaging | T | T | 19.48 | 0.385 | 3.668 | 0.00003520 |
| 5 | TOF | | c.5776C>T | p.Arg1926Cys | | Yes | deleterious | probably damaging | T | D | 32 | 0.465 | 7.863 | 0.0001286 |
| 6 | PS | | c.5776C>T | p.Arg1926Cys | | Yes | deleterious | probably damaging | T | D | 32 | 0.465 | 7.863 | 0.0001286 |
| 7 | TGA, CoA | | c.5189C>T | p.Pro1730Leu | | Yes | tolerated | benign | T | T | 17.77 | 0.036 | 2.719 | 0.0002602 |
| 8 | HLH | | c.4049G>A | p.Arg1350His | | Yes | tolerated | benign | T | T | 12.39 | 0.219 | 2.089 | 0.00007650 |
| 9 | TOF | | c.2477G>A | p.Cys826Tyr | | Yes | deleterious | probably damaging | D | D | 26.2 | 0.903 | 7.305 | absent |
| 10 | TGA/VSD | | c.6130G>A | p.Ala2044Thr | | Yes | tolerated | possibly damaging | T | T | 19.84 | 0.3 | 5.669 | 0.00005949 |
| 11 | HLH | | c.3190G>A | p.Asp1064Asn | | Yes | tolerated | probably damaging | T | T | 24.7 | 0.451 | 2.9 | 0.00003286 |
| 12 | Heterotaxy | | c.4426G>A | p.Gly1476Ser | | Yes | deleterious | possibly damaging | D | D | 25.3 | 0.809 | 5.581 | 0.00002356 |
| 13 | TGA | | c.1136A>G | p.Asn379Ser | | Yes | tolerated | benign | T | T | 19.46 | 0.352 | 7.47 | 0.000009595 |
| 14 | TOF | | c.2218G>T | p.Asp740Tyr | | Yes | tolerated | possibly damaging | D | T | 17.58 | 0.648 | 0.763 | 0.000001369 |
| 15 | BAV, aortopathy | | c.7397C>T | p.Thr2466Met | | Unknown | deleterious | possibly damaging | T | T | 23 | 0.26 | 5.099 | 0.0001381 |
| 16 | Arrhythmia | | c.4325C>G | p.Pro1442Arg | | Unknown | deleterious | possibly damaging | T | D | 25.1 | 0.504 | 7.533 | 0.000002058 |
| 17 | HCM | | c.7363A>T | p.Thr2455Ser | | Unknown | tolerated | benign | T | T | 15.34 | 0.295 | 3.658 | 0.000002078 |
| 18 | Aortopathy | | c.3068A>G | p.Asn1023Ser | | Unknown | deleterious | benign | D | D | 22.6 | 0.713 | 7.963 | 0.000008548 |
| 19 | HCM | | c.3061G>A | p.Asp1021Asn | | Unknown | tolerated | benign | T | T | 21.1 | 0.247 | 4.056 | 0.00002504 |
