# Supplementary material for: Expanding the phenotypic spectrum of NOTCH1 variants: clinical manifestations in families with congenital heart disease
Source: Eur J Hum Genet. 2024 May 22;32(7):795–803. doi: 10.1038/s41431-024-01629-4 (PMC11219983; doi:10.1038/s41431-024-01629-4)
Supplement: Supplementary file 4 — Supplementary Table 4 [file 41431_2024_1629_MOESM4_ESM.docx]

**Supplementary Table 4:** Clinical features of all consented individuals with *NOTCH1* variants. Abbreviations include: ADHD = attention deficit hyperactivity disorder; ASD = atrial septal defect; AVM = arteriovenous malformation; BAV = bicuspid aortic valve; CHD = congenital heart disease; CMA = chromosomal microarray; CT = computed tomography; DORV = double outlet right ventricle; EFE = endocardial fibroelastosis; FV = familial variant genetic test; GA = gestational age; GP = gene panel; GS = genome sequencing; HLH = hypoplastic left heart; LA = left atrium; LPA = left pulmonary artery; LPV = left pulmonary vein; LV = left ventricle; MAPCA = major aortopulmonary collateral arteries; MPA = main pulmonary artery; MRA = magnetic resonance angiography; MRI = magnetic resonance imaging; NA = not assessed (when referring to physical exam, means no in-person physical exam was performed); NR = not recorded; PA = pulmonary artery; PDA = patent ductus arteriosus; RPA = right pulmonary artery; RPV = right pulmonary vein; RV = right ventricle; RVOT = right ventricular outflow tract; SV = single variant genetic test; SVC = superior vena cava; TAPVC = total anomalous pulmonary venous connection; TGA = transposition of the great arteries; TOP = termination of pregnancy; TOF = tetralogy of Fallot; U/S = ultrasound; VSD = ventricular septal defect; VUS = variant of uncertain significance.

| **Patient**  [age] | **Genetic Results**  [test performed] | **Cardiothoracic Features**  [test performed, *if applicable;* age] | **Physical Features**  [age at exam] | **CNS Features**  [test performed; age] | **Neurodevelopmental Features**  [age at formal assessment] | **Ophthalmologic Features**  [age at exam] | **Abdominal Features**  [test performed; age] | **Other Features**  [age at presentation] | **Additional Relevant Family History** |
| --- | --- | --- | --- | --- | --- | --- | --- | --- | --- |
| **Family A** | | | | | | | | | |
| **A-III:4**  [6.9yr] | **[GS] –** *NOTCH1* 9q34.3del (pathogenic) | **[Echo; 0d] –** TOF; pulmonary atresia  **[MRI; 4.6m]** – MPA aneurysm | **[3.4yr] –**  **Scalp:** Normal  **Limbs:** Normal  **Skin:** Reversible cutis marmorata | **[Brain MRI; 4.1yr] –**Subtle increased signal in left hippocampus; small anterior pituitary gland; increased signal in cerebral white matter  **[Brain MRA; 4.1yr] –** Normal | NA (normal by parental report) | NA | [**Abdominal U/S; 4.1yr] –** Normal liver, kidneys, gallbladder, spleen, bladder, bowels. Portal veins, pancreas not imaged. | **[4yr] –** Seizures | **[A-I:2]** – TOF (no genetic testing)  **[A-I:4]** – VSD (no genetic testing)  **[A-III:2]** – “hole in the heart” (no genetic testing) |
| **A-III:6**  [TOP] | **[CMA] –** *NOTCH1* 9q34.3del (pathogenic) | **[Fetal Echo; 19w GA] –** HLH variant (mitral atresia, DORV); hypoplastic aorta | **[Autopsy; 21w GA] –**  **Scalp:** Normal  **Limbs:** Normal  **Skin:** NA | **[Anatomy Scan; 19w GA] –** Normal brain, normal skull shape | NA | NA | **[Anatomy Scan; 19w GA] –** Hyperechogenic bowel; normal kidneys, bladder | **[Autopsy; 21w GA]** –Coronal clefts L3, L4 |  |
| **A-II:4**  [39.8yr] | **[CMA] –** *NOTCH1* 9q34.3del (pathogenic) | **[Echo; age NR]** – Perimembranous VSD; secundum ASD | NA (no concerning features by self-report) | NA | NA (self-reported learning disability) | NA | NA | **[6yr] -** Hydrocele |  |
| **Family B** | | | | | | | | | |
| **B-II:1**  [9.6yr] | **[CMA]** – Uninformative  **[GS] –** *NOTCH1*  c.13_14dupCT  (likely pathogenic) | **[Echo; 0d] –** Tricuspid atresia; VSD; normally related great arteries **[Echo; 2.5yr] –** Bilateral pulmonary AVMs | **[7.1yr] –**  **Scalp:** Normal  **Limbs:** Short 5^th^ fingers bilaterally and short thumbs  **Skin:** 5cm vascular malformation on back | **[Brain MRI; 8.8yr] –** Normal  **[Brain MRA; 8.8yr] –** Normal | **[3.5yr]** – Delayed speech articulation | **[9.3yr] –** Refractive error | **[Liver U/S with Doppler; 6.3yr] –** Nonspecific periportal echoes; elevated hepatic elastography  **[Kidney U/S with Doppler; 7.6yr] –** Normal  [**Abdominal U/S; 7.6yr] –** Normal portal veins, gallbladder, pancreas, spleen, bladder, bowels | **[3.8yr] –** Unexplained abdominal pain | None |
| **Family C** | | | | | | | | | |
| **C-III:1**  [17.4yr] | **[SV, GS] –** *NOTCH1*  c.2995G>A  (VUS) | **[Echo; 0d] –** Severe aortic stenosis; BAV; mitral stenosis  **[MRI; 12.9yr] –** LV EFE | **[14.0yr] –**  **Scalp:** Normal  **Limbs:** 5^th^ finger clinodactyly  **Skin:** Cutis marmorata | **[Brain MRI; 16.6yr] –** Post-ischemic areas. Scattered tiny foci of susceptibility in the supratentorial and infratentorial brain, likely related to residua of remote prior microhemorrhages.  **[Brain MRA; 16.6yr] –** Normal | NA (normal by parental report) | NA | **[Liver U/S with Doppler; 16.5yr] –** Persistent atrophy of the left lobe of the liver, in keeping with sequela of previous occlusive left portal vein thrombosis.  **[Kidney U/S with Doppler; 16.5yr] –** Normal  [**Abdominal U/S; 16.5yr] –** Smallc calcification in relation to the wall of the main portal vein that may represent sequela of previous mural thrombus. Normal gallbladder, pancreas, spleen, bladder. | None | None |
| **C-III:3**  [TOP] | **[FV, GS] –** *NOTCH1*  c.2995G>A  (VUS) | **[Fetal Echo; 20w GA] –** HLH (mitral and aortic atresia); hypoplastic aorta | **[Autopsy] –**  **Scalp:** Normal  **Limbs:** Bilateral finger clinodactyly  **Skin:** NA | NA | NA | NA | **[Autopsy]** – Liver calcifications, some involving degenerating hepatocytes. Kidneys, spleen, bladder, bowels normal. | **[Autopsy]** – Hypertelorism; down-slanting palpebral fissures; infraorbital creases |  |
| **C-II:1**  [43.9yr] | **[FV, GS] –** *NOTCH1*  c.2995G>A  (VUS) | **[Echo; age NR]** – Severe aortic stenosis, BAV  **[Echo, 39.4yr]** – Mildly dilated ascending aorta | NA (no concerning features by self-report) | **[Brain MRI; 43.2yr] –** Normal | NA (normal by self-report) | **[43.3yr] –** Normal | **[Liver U/S with Doppler; 42.9yr] –** Homogeneous parenchymal echogenicity. Normal Doppler.  **[Kidney U/S with Doppler; 42.9yr] –** 7mm cortical cyst in the left upper renal pole. Normal Doppler.  **[Portal Vein U/S with Doppler; 42.9yr] –** Normal Doppler.  [**Abdominal U/S; 42.9yr] –** Normal gallbladder, pancreas, spleen. | None |  |
| **C-I:2**  [68.3yr] | **[FV] –** *NOTCH1*  c.2995G>A  (VUS) | **[CT Thorax, 66.0yr] –** Coronary artery atherosclerosis  **[Echo, 66.1yr] –** Normal | NA (vascular malformation on hand; no other concerning features by self-report) | **[Brain MRI; 63.1yr] –** Chronic microangiopathic ischemia | NA (normal by self-report) | NA | **[CT Abdomen/Pelvis, 66.0yr] –** Small hiatus hernia; 7mm hepatic lesion; hepatic steatosis. | **[66.0yr] –** Scoliosis  **[62.3yr]** – Giant cell arteritis/poly myalgia rheumatica overlap |  |
| **Family D** | | | | | | | | | |
| **D-II:2**  [22.2yr] | **[GS] –** *NOTCH1*  c.141-1G>C (likely pathogenic) | **[Echo; 10d]** – Common arterial trunk; right aortic arch | **[20.9yr] –**  **Scalp:** Normal  **Limbs:** Normal  **Skin:** Normal | **[Brain MRI; 21yr] –** Normal.  **[Brain MRA; 21yr] –** Normal. | NA (normal by parental/self-report) | **[21yr] –** Normal. | **[Liver U/S with Doppler; 21yr] –** Normal.  **[Kidney U/S with Doppler; 21yr] –** Normal.  [**Abdominal U/S; 21yr] –** Normal. | None | **[D-II:1] –** TGA (no genetic testing) |
| **D-II:3**  [18.7yr] | **[GS] –** *NOTCH1*  c.141-1G>C (likely pathogenic) | **[Echo; age NR]** – TOF with pulmonary atresia and MAPCAs | **[14.9yr] –**  **Scalp:** Normal  **Limbs:** Normal  **Skin:** Normal | **[Brain/Neck MRI; 13.9yr] –** Normal  **[Brain/Neck MRA; 13.9yr] –** Bilateral hypoplastic vertebrobasilar arterial system (left worse than right) | NA (gross and fine motor delay, learning disability, and short-term memory loss by parental report) | **[17.5yr]** – Normal | **[Liver U/S with Doppler; 17.6yr] –** Echogenic echotexture of the liver, in keeping with fatty infiltration.  **[Kidney U/S with Doppler; 17.6yr] –**Normal sized kidneys and normal Doppler assessment. Poor renal corticomedullary differentiation.  [**Abdominal U/S; 17.6yr] –** Minimal splenomegaly. Normal portal veins, gallbladder, bladder, bowels. | None |  |
| **Family E** | | | | | | | | | |
| **E-III:1**  [3.7yr] | **[CMA]** – Normal  **[GS] –** *NOTCH1*  c.568C>T (VUS) | **[Echo; 0d] –** TOF; crossed small pulmonary arteries | **[1.2yr] –**  **Scalp:** Normal  **Limbs:** Normal  **Skin:** Normal | **[Brain/Neck MRI; 2.9yr] –** A small focus of FLAIR/T2 hyperintensity in the right frontal white matter and tiny focus in the right parietal subcortical white matter are nonspecific but may represent foci of prior ischemia. Scattered microhemorrhages likely related to previous cardiac surgery.  **[Brain/Neck MRA; 2.9yr] –** Tiny focal outpouching involving the proximal A2 segment of the right anterior cerebral artery, which may represent a tiny aneurysm or infundibulum. | NA (normal by parental report) | NA | **[Liver U/S with Doppler; 2.9yr] –** Normal  **[Kidney U/S with Doppler; 2.9yr] –**Normal  [**Abdominal U/S; 2.9yr] –** Normal | None | None |
| **E-II:2**  [26.4yr] | **[CMA]** – Normal  **[GS] –** *NOTCH1*  c.568C>T (VUS) | **[Echo; age NR]** – TOF; LPA stenosis; pulmonary regurgitation  **[ECG; age NR]** – ventricular tachycardia | NA (no concerning features by self-report) | NA | NA (normal by self-report) | NA | NA | **[age NR]** – Scoliosis; asthma |  |
| **E-II:3**  [23.4yr] | **[CMA]** – Normal  **[GS] –** *NOTCH1*  c.568C>T (VUS) | **[Echo; age NR]** – TOF | NA (no concerning features by self-report) | NA | NA | NA | NA | NA |  |
| **E-II:4**  [23.4yr] | **[CMA]** – Normal  **[GS] –** *NOTCH1*  c.568C>T (VUS) | **[Echo; age NR]** – TOF | NA (no concerning features by self-report) | NA | NA | NA | NA | NA |  |
| **E-I:2**  [54.0yr] | **[CMA]** – Normal  **[GS] –** *NOTCH1*  c.568C>T (VUS) | NA | NA (no concerning features by self-report) | NA | NA | NA | NA | NA |  |
| **Family F** | | | | | | | | | |
| **F-III:1**  [5.7yr] | **[SV, GS] –** *NOTCH1* c.5814C>G (likely pathogenic) | **[Echo; 0d]** – HLH variant (hypoplastic LV; DORV; mitral stenosis); severe coarctation; hypoplastic aortic arch | NA (mottling; no other concerning features by parental report) | **[Brain MRI; 4.6yr] –** Diffusely small corpus callosum; mild prominence of lateral ventricles with slightly reduced cerebral white matter volume; scattered hyperintensities in both cerebral hemispheres.  **[Brain MRA; 4.6yr] –** Normal | **[3.0yr]** – Gross motor delay; selective mutism | NA | **[Kidney U/S with Doppler; 4.6yr] –** Normal | **[2.9yr] –** Hypertension (unknown etiology)  **[3.0yr] –** Right leg bowing | **[F-I:2] –** BAV (negative for familial *NOTCH1* variant)  [**F-III:2**] – Right aortic arch (negative for familial *NOTCH1* variant) |
| **F-II:2**  [35.4yr] | **[FV, GS] –** *NOTCH1* c.5814C>G (likely pathogenic) | **[Echo; 34.6yr]** – Mild biventricular dilatation, mild aortic root dilatation (4.1cm), mild aortic regurgitation, cannot rule out bicuspid aortic valve | NA (no concerning features by self-report) | **[Brain MRI; 34.7yr] –** Suspected focus of subependymal grey matter heterotropia in the left frontal region  **[Brain MRA; 34.7yr] –** Normal | NA (Normal by self-report) | NA | [**Abdominal U/S; 28.7yr] –** Hyperechoic nodule in right lobe of liver; normal kidneys, gallbladder, spleen. Bladder, pancreas, bowels not visualized. | **[Congenital]** – Poland anomaly (absent right chest muscle) |  |
| **F-I:1**  [66.6yr] | **[FV] –** *NOTCH1* c.5814C>G (likely pathogenic) | **[Echo; 57.4yr] -** BAV  **[ECG; 57.4yr] -** Non**-**ST-elevation myocardial infarction  **[Echo; 67.4yr]** - moderate-severe ascending aortic aneurysm (4.8 cm) and mild dilation of aortic root (4.0 cm)  Self-reported history of heart murmur in the newborn period and septal defect that closed spontaneously. | NA (no concerning features by self-report) | NA | NA (normal by self-report) | [**61.1yr**] - Bilateral optic nerve cupping; Myopia -3 | NA | None |  |
| **F-II:1**  [33.8yr] | **[FV] –** *NOTCH1* c.5814C>G (likely pathogenic) | **[Echo; 32.3yr]** - Normal | NA (no concerning features by self-report) | NA | NA (normal by self-report) | NA | NA | None |  |
| **Family G** | | | | | | | | | |
| **G-II:1**  [2.6yr] | **[CMA]** – Uninformative  **[GS] –** *NOTCH1*  c.3654T>A  (pathogenic) | **[Echo; 1d] –** HLH variant (mitral atresia, DORV); VSD; mixed supracardiac TAPVC (RPV to LA, LPV drain to a vertical vein which drains to the innominate vein), small LA with multiple fenestrations in the atrial septum | **[7.5mo] –**  **Scalp:** Normal  **Limbs:** Normal  **Skin:** Mottling | **[Brain MRI; 3.2mo] –** Microcephaly (head circumference 32cm, 2^nd^ percentile); enlarged pericerebral extra-axial spaces and lateral ventricles; thin corpus callosum, midbrain accessory commissure; elongated medulla; large left parietal cephalohematoma; white matter diffusion in keeping with early subacute infarction; subgleal hematoma; diffusely delayed myelin maturation.  **[Brain MRA; 3.2mo]** – Normal | **[1.1yr] –** Normal | **[7.9mo] –** Normal | **[Liver U/S with Doppler; 7.7mo] –** Enlarged liver; nonspecific periportal echoes; mildly increased hepatic velocities.  **[Kidney U/S with Doppler; 7.7mo] –** Normal  **[Portal Vein U/S with Doppler; 7.7mo] –** Small portal venous system with nonspecific slow flow in left portal vein  [**Abdominal U/S; 7.7mo] –** Normal gallbladder, pancreas, spleen, bowel; bladder not visualized | **[4.6mo] –** Seizures | None |
| **Family H** | | | | | | | | | |
| **H-III:1**  [16.9yr] | **[GP] –** *NOTCH1* c.4415G>A  (VUS) | **[Echo; 1d] –** TOF; right aortic arch; discontinuous pulmonary arteries with the left pulmonary artery arising from a left sided arterial duct from the base of the left innominate artery | **[12.2yr] –**  **Scalp:** Cutis aplasia  **Limbs:** Finger brachydactyly; short left 2/3 toes with hypoplastic nails  **Skin:** Café-au-lait spot (2cm, back of right hand) | NA | NA (normal by parental report) | NA | NA | **[Congenital]** – Solitary R kidney | **[H-II:4] –** “CHD” (no genetic testing)  **[H-II:5] –** Cutis aplasia (no genetic testing)  **[H-II:6] –** Cutis aplasia (no genetic testing)  **[H-II:7] –** TOF, cutis aplasia (reportedly positive for familial *NOTCH1* variant)  **[H-III:5] –** Cutis aplasia (no genetic testing) |
| **H-III:3**  [11.5yr] | **[FV] –** *NOTCH1* c.4415G>A  (VUS) | **[Echo; 0d] –** Severe aortic stenosis; BAV; mild-moderate mitral stenosis | **[6.8yr] –**  **Scalp:** Cutis aplasia; bony defect in parietal bone  **Limbs:** Finger brachydactyly  **Skin:** Abdominal hyper-pigmentation | **[Brain MRI; 1d] –** Normal | NA (normal by parental report) | NA | NA | None |  |
| **H-II:2**  [50.1yr] | **[FV] –** *NOTCH1* c.4415G>A  (VUS) | NA | **[45.4yr] –**  **Scalp:** Cutis aplasia  **Limbs:** Normal  **Skin:** Normal | NA | NA (normal by self-report) | NA | NA | None |  |
| **Family I** | | | | | | | | | |
| **I-III:2**  [d. 6m] | **[FV] –** *NOTCH1* c.4579C>T (pathogenic) | **[Echo, 1d] –** DORV, mitral valve atresia, severely hypoplastic LV, BAV, aortic coarctation, atretic coronary sinus.  **[ECG, 2w]** – Supraventricular tachycardia.  **[Autopsy] –** Single posterior coronary artery ostium, persistent left SVC with stenosis draining into markedly dilated coronary sinus. | **[Autopsy] –**  **Scalp:** Normal  **Limbs:** Normal  **Skin:** Normal | **[Brain MRI; 2d] –** White matter injury (right corona radiata, bilateral petriogonal); bilateral postero-lateral thalamic injury; hypoplastic left transverse sinus, fenestration of superior sagittal sinus.  **[Brain MRA; 2d] –** Hypoplastic right vertebral artery | NA | NA | [**Abdominal U/S; 3d] –** Edema of gallbladder wall (secondary to cardiac condition), small spleen. Normal liver, pancreas, bowel, kidneys. | **[6m] –** Large left middle cerebral artery ischemic infarct.  **[6m]** – Subclinical seizures  **[Autopsy] –** Pulmonary hypertension | None |
| **I-III:3**  [2.9yr] | **[FV] –** *NOTCH1* c.4579C>T (pathogenic) | **[Echo, 5m] –** Normal | **[1.1yr] –**  **Scalp:** Cutis aplasia  **Limbs:** Normal  **Skin:** Cutis marmorata; 2x café-au-lait spots | **[Brain MRI; 3.4mo] –** Normal | NA (normal by parental report) | **[2.6yr] –** Tortuous blood vessels in the fundus. | **[Liver U/S with Doppler; 1.8yr] –** Normal  **[Kidney U/S with Doppler; 1.8yr] –** Normal  **[Portal Vein U/S with Doppler; 1.8yr] –** Normal  [**Abdominal U/S; 1.8yr]** – Normal gallbladder, pancreas, spleen, bladder; bowels not visualized. | None |  |
| **I-II:3**  [37.2yr] | **[GP] –** *NOTCH1* c.4579C>T (pathogenic) | **[Echo, 24.1yr] –** Mildly dysplastic pulmonary valve and trivial pulmonary stenosis  **[Echo, 35.8yr] –** BAV | **[34.0yr] –**  **Scalp:** Normal  **Limbs:** Normal  **Skin:** Normal | NA | NA (normal by self-report) | NA | NA | None |  |
| **I-III:1**  [12.5yr] | **[FV] –** *NOTCH1* c.4579C>T (pathogenic) | **[Echo, 1d] –** DORV-TOF type; small confluent pulmonary arteries; dysplastic BAV; mild aortic regurgitation  **[Echo, 6.7yr] –** BAV | NA (no concerning features by self-report) | NA | **[18m] –** Expressive language delay  **[9yr] –** ADHD, moderate learning disability | NA | NA | None |  |
| **I-II:2**  [d. 36yr] | **Obligate carrier –** *NOTCH1* c.4579C>T (pathogenic) | **[Echo, age NR]** – VSD, BAV, aortic stenosis, subaortic stenosis, moderate aortic insufficiency, pulmonary stenosis, mild pulmonary insufficiency.  **[ECG, 35.6yr] –** frequent ventricular ectopy, including non-sustained ventricular tachycardia. | NA | NA | NA (normal by self-report) | NA | NA | **[23.6yr] –** Mild hypertension  **[28.0yr] –** Eosinophilic esophagitis |  |
| **I-I:2**  [62.7yr] | **[FV] –** *NOTCH1* c.4579C>T (pathogenic) | **[Echo, 15yr] –** Pulmonary stenosis  **[Echo, 60.5yr] –** BAV; severe aortic stenosis; moderate aortic regurgitation; severe aortic calcification | NA (no concerning features by self-report) | NA | NA | NA | NA | None |  |
| **Family J** | | | | | | | | | |
| **J-II:2**  [2.5yr] | **[GS] –** *NOTCH1* c.866-2A>G (likely pathogenic) | **[Echo, 1d] –** TOF with pulmonary atresia, MAPCAs (4), absent central PA, right aortic arch, retro-aortic innominate vein. | **[5.9m] –**  **Scalp:** Normal  **Limbs:** Normal  **Skin:** Normal | **[Brain MRI; 3.2yr] –** A few nonspecific scattered foci of non-enhancing T2/FLAIR hyperintensities in the left frontal lobe and corona radiata. Numerous foci of susceptibility artifact, likely related to previous cardiac procedure.  **[Brain MRA; 3.2yr]:** Tortuosity of the cervical internal carotid arteries bilaterally at the skull base. | **[5.9m] –** Normal | NA | **[Liver U/S with Doppler; 2.6yr] –** Normal  **[Kidney U/S; 2.6yr] –** Normal  **[Portal Vein U/S 2.6yr] –** Normal  [**Abdominal U/S; 1.8yr]** – Normal gallbladder, pancreas, spleen, bladder, bowels. | None | None |
| **J-I:1**  [34.6yr] | **[GS] –** *NOTCH1* c.866-2A>G (likely pathogenic) | **[Echo, 33.6yr] –** small PDA, moderate RV dilatation (partial anomalous pulmonary venous drainage not excluded) with mildly reduced systolic function out-of-keeping with PDA shunt, dilated RA, dilated MPA/LPA/RP. | NA (no concerning features by self-report) | **[Brain MRI; 34.6yr] –** Left parietal deep white matter old small lacunar infarct or dilated perivascular space. No evidence of acute intracranial abnormality.  **[Brain MRA; 34.6yr] –** Normal. | NA | NA | NA | **[33.6yr] –** Pulmonary hypertension. |  |
| **Family K** | | | | | | | | | |
| **K-II:1**  [5.3yr] | **[GS]** - *NOTCH1* c.5349del (likely pathogenic) | **[Echo, 5m] -** Small to moderate perimembranous VSD  **[Echo, 1.5yr] -** Criss-crossed branch pulmonary arteries  **[Echo, 1.6yr] -** Severely dilated right coronary artery, ostial atresia of the left main coronary artery | NA (possible café-au-lait macule on back; no other concerning features by self-report). | NA | NA (normal by parental report) | **[5.3yr]** - Normal | NA | None |  |
| **K-I:2**  [35.0yr] | **[GS]** - *NOTCH1* c.5349del (likely pathogenic) | **[Echo, 35yr]** - Normal | NA (bald spots on scalp, suspected cutis aplasia no other concerning features by self-report). | **[Brain MRI; 35yr]** - Normal | NA (normal by self-report) | **[35yr] -** Normal | NA | None |  |
